# Intravital imaging of the functions of immune cells in the tumor microenvironment during immunotherapy

**DOI:** 10.3389/fimmu.2023.1288273

**Published:** 2023-12-06

**Authors:** Xuwen Peng, Yuke Wang, Jie Zhang, Zhihong Zhang, Shuhong Qi

**Affiliations:** ^1^ Britton Chance Center for Biomedical Photonics, Wuhan National Laboratory for Optoelectronics-Huazhong University of Science and Technology, Wuhan, Hubei, China; ^2^ MoE Key Laboratory for Biomedical Photonics, School of Engineering Sciences, Huazhong University of Science and Technology, Wuhan, Hubei, China

**Keywords:** intravital imaging, tumor microenvironment, immune cells, spatio-temporal information, movement behavior, cancer immunotherapy

## Abstract

Cancer immunotherapy has developed rapidly in recent years and stands as one of the most promising techniques for combating cancer. To develop and optimize cancer immunotherapy, it is crucial to comprehend the interactions between immune cells and tumor cells in the tumor microenvironment (TME). The TME is complex, with the distribution and function of immune cells undergoing dynamic changes. There are several research techniques to study the TME, and intravital imaging emerges as a powerful tool for capturing the spatiotemporal dynamics, especially the movement behavior and the immune function of various immune cells in real physiological state. Intravital imaging has several advantages, such as high spatio-temporal resolution, multicolor, dynamic and 4D detection, making it an invaluable tool for visualizing the dynamic processes in the TME. This review summarizes the workflow for intravital imaging technology, multi-color labeling methods, optical imaging windows, methods of imaging data analysis and the latest research in visualizing the spatio-temporal dynamics and function of immune cells in the TME. It is essential to investigate the role played by immune cells in the tumor immune response through intravital imaging. The review deepens our understanding of the unique contribution of intravital imaging to improve the efficiency of cancer immunotherapy.

## 1 Introduction

In recent decades, cancer immunotherapy has made significant progress and is regarded as a breakthrough in cancer therapy (1, 2). When the immune response was triggered by immunotherapy in the tumor microenvironment (TME), both innate and adaptive immune cells were activated to eliminate the tumor (3, 4). Nevertheless, the TME is still like a “black box”. Scientists know that the input information is “immunotherapy” and the output information is “tumor elimination or tumor growth” (5), but the understanding of the immune cell spatio-temporal changes in the TME is still limited. To understand the mechanisms of cancer immunotherapy, it is crucial to visualize the functional information of immune cells (6), including their morphology, distribution, movement, migration, recruitment and interaction with other cells.

The immune response involves the integration of spatio-temporal information from numerous immune molecules and immunocytes *in vivo* (7, 8). To comprehensively illustrate the essence of the immune response, it is necessary to visualize the structure, distribution, interaction, function and dynamic changes of molecules and cells. Research instruments that have high spatio-temporal resolution, prolonged dynamic monitoring capabilities, multi-parallel functionality, and 4D detection are necessary (9). Intravital molecular imaging has been considered as the most suitable technique for monitoring the mobility and function of various immune molecules and cells in the TME (7, 8, 10).

Since 2002, intravital imaging systems such as multiphoton and confocal microscopy have been employed to study the immune system, such as analyzing cellular motility, cell distribution, and cell interaction, etc. (11, 12). The professor Zhihong Zhang’s group has abundant foundations in the field of intravital imaging for studying tumor immune response (5, 13–16). This review provides a concise overview of the workflow of intravital imaging, multi-color labeling strategies, imaging windows and methods of intravital imaging data analysis based on the previous research of Professor Zhang’s group. It also summarized the latest research in utilizing intravital imaging to investigate the immune cell function in the TME during immunotherapy. The review serves as guidelines for researchers about how to apply intravital imaging technology into their researches and develop a deeper understanding of the roles played by immune cells in the cancer immunotherapy.

## 2 Construction of the intravital imaging model

**Figure 1:**
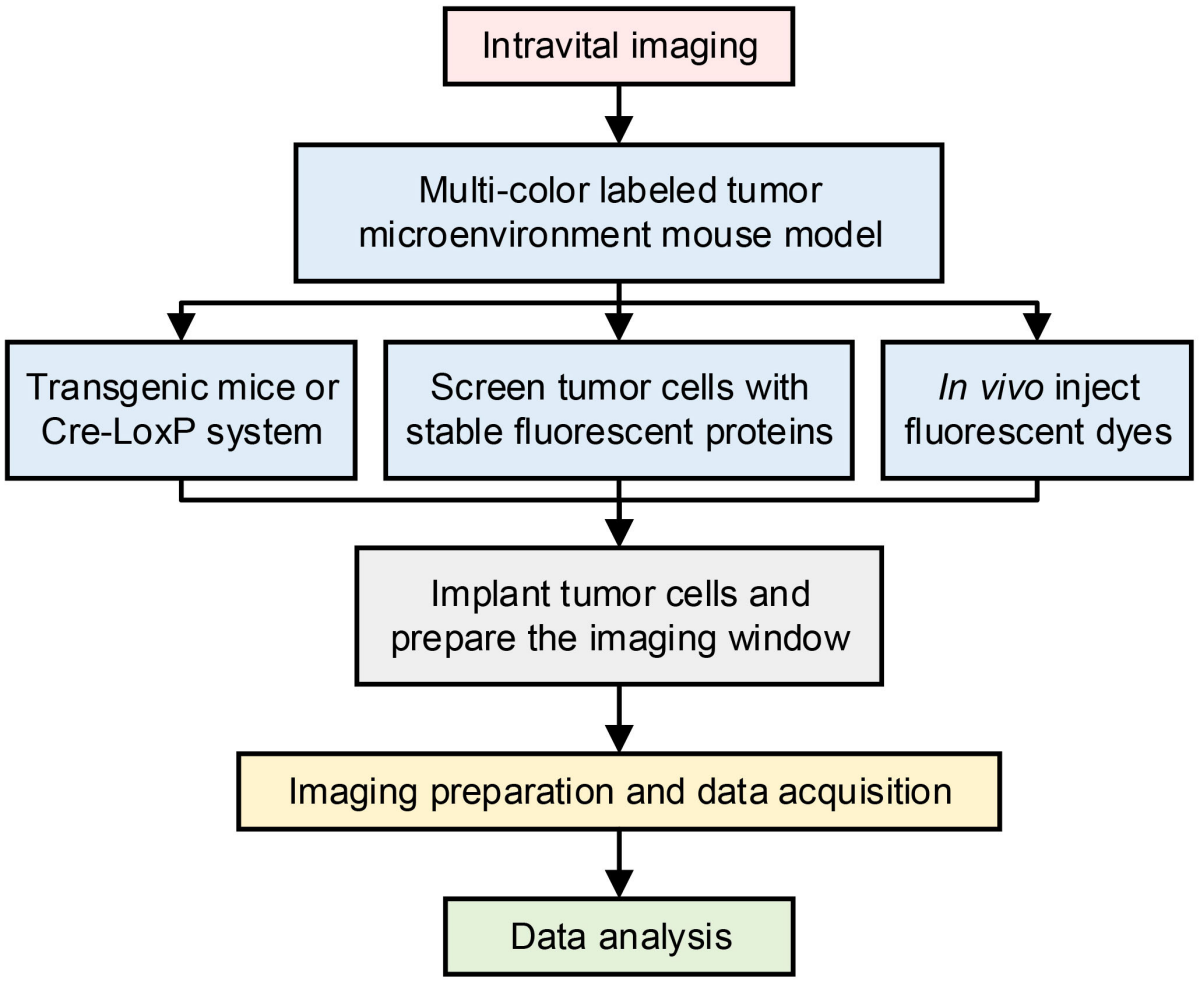
Workflow for intravital imaging.

Firstly, we would like to describe the basic process of intravital imaging (**Figure 1**) and the protocol for constructing multicolor imaging mice models to explore the TME *in vivo*.

### 2.1 Workflow for intravital imaging

(1) Build a multi-color labeled TME mouse model: 1) Screen tumor cell lines that stably express fluorescence proteins *in vitro*. 2) Use transgenic mice or the Cre-LoxP system to generate certain types of immune cells labeled with fluorescence protein. 3) Implant screened tumor cell lines into the transgenic mice.
(2) Prepare the imaging window: According to tumor models on different organs, select the appropriate imaging window. Prepare the imaging window, and expose the tumor tissues to the intravital imaging system.
(3) Imaging preparation and data acquisition: 1) Temperature, anesthetic state, and oxygen maintenance of the imaging mice; 2) Inject fluorescent dye to label blood vessels or other subsets of immune cells, etc. to establish a mouse model with multi-color labeled TME. 3) Choose the suitable imaging system according to the position of the imaging windows, including choosing upright or inverted microscopy, and choosing confocal microscopy or multi-color microscopy, etc.; 4) Select the appropriate imaging objectives, laser wavelengths, filters and channels; 5) Set appropriate imaging parameters according to the imaging requirements, such as scanning speed, 3D imaging, imaging interval time, etc.
(4) Data analysis: After acquiring the original imaging data, a series of analysis software can be used to analyze the imaging data, and obtain information such as cell spatial distribution, cell morphology, and movement behavior.

### 2.2 Labeling methods

#### 2.2.1 Fluorescent dyes

Here is the guideline for selecting a fluorescent dye. 1) Excitation and emission spectra should be separated as far as possible, allowing intravital imaging of different cells at the same time. We can choose Brilliant Violet™ 421 (Excitation maximum (Ex): 405 nm/Emission maximum (Em): 421 nm) to label the first type of immune cells, CFSE (Ex: 500 nm/Em: 520 nm) to label the second type of immune cells, and Dye eFluor® 670 (Ex: 647 nm/Em: 670 nm) to label the third type of immune cells. 2) There are also some cell localization dyes (DAPI (Ex: 350 nm/Em: 470 nm) staining for the nucleus, DiI (Ex: 549 nm/Em: 565 nm) staining for cell membrane, MitoTracker™ Red CMXRos (Ex: 579 nm/Em: 599 nm) staining for mitochondrion, etc.), which can be selected according to the imaging requirements (17). 3) Choose the dye which is uneasy to be bleached, has a long retention time and low toxicity. We also could use a few website tools to make a reasonable choice based on the characteristics of the dye. Here is a list of some website tools for selecting Fluorescent Dyes (**Table 1**).

**Table 1:** Commonly used website tools for selecting Fluorescent Dyes.

Tool	Characteristic	Website
Fluorescence Spectraviewer	(1) Directly show the excitation and emission plot on the graph; (2) Display the information of multiple dyes at the same time; (3) Alternatively add laser sources and emission filters.	(18)
Spectra Viewer	(1) Display the information of up to15 dyes at the same time; (2) Model certain spectral simulations.	(19)
FPbase Spectra Viewer	A fluorescent protein database contains 887 proteins and 948 fluorescence protein spectra.	(20)
Two-photon excitation fluorescence suitable dyes Viewer	(1) This tool provides a list of two-photon probes as well as a matching wavelength range; (2) There are 280 kinds of two-photon probes.	(21)

#### 2.2.2 Nanoparticles

Fluorescent semiconductor nanocrystals (quantum dots) or fluorophores can be incorporated into organic or inorganic nanoparticles through embedding, covalent bonding, and supramolecular assembly. These nanoparticles are specifically designed to label particular immune cell types, including macrophages (22), monocytes (16), neutrophils (23), T cells (24), etc. This allows for precise identification and tracking of these immune cells based on the unique characteristics of the nanoparticles.

#### 2.2.3 Transgenic mice

Transgenic mice models, such as those using the Cre-LoxP system and CRISPR/Cas9 gene editing technology, have greatly advanced research in immunology. These models enable the labeling of specific immune cells with fluorescent proteins for visualization and research. We have listed different stains of transgenic mice (**Table 2**).

**Table 2:** Commonly used transgenic mice with immune cells labeled with the fluorescent protein.

Immune cell	Transgenic mice	Characteristic	Refs
Monocyte	CCR_2_-RFP	Marked approximately 86% of Ly6C^high^ monocytes and 18% of Ly6C^low^ monocytes, 85~95% of natural killer cells (NKs) and 5~10% T cells	(25)
	CX_3_CR_1_-GFP	Marked dendritic cells (DCs), monocytes, microglia, and NK cells through the endogenous Cx3cr1 locus	(26)
DC	CD11c- EYFP	The subtype of DC that could be bright enough to be visualized is CD11c^+^CD19^-^CD3^-^CD86^+^ MHC-II^+^ DC.	(27)
	CD11c-Cre-GFP	Marked majority of DC, all CD11c^high^CD11b^+^ cells, marked plasmacytoid DCs (pDCs, defined as PDCA-1^+^CD11c^low^B220^+^)	(28)
Neutrophil	LysM-EGFP	Marked myelomonocytic cells: macrophages and neutrophil granulocytes Peripheral blood: marked 14~44% of leukocytes Bone marrow: marked the myelomonocytic lineage	(29)
	CatchupIVM-red	Marked neutrophils strongly and specifically, also marked few eosinophils and basophils Spleen and peripheral blood: marked >90% neutrophils Peripheral blood: marked 90% of CD11b^+^Ly6G^+^ cells Bone marrow: all marked neutrophil cells were CD11b^+^ Ly6G^+^	(30)
T cell	CXCR6-GFP	Marked liver leukocytes, live NKT, CD41^+^ T cell, CD81^+^ T cell, some γδT and CD161^+^ NK cells, CD31^+^ T cells in lymph nodes and spleen and few NK cells	(31)
γδT	γδT-GFP	Small intestines: marked CD3ϵ^+^ TCRγδ^+^ intraepithelial lymphocytes (IELs) Spleen and lymph nodes: TCRγδ cells 5% of total thymocytes and all TCRγδ thymocytes, double-negative cells (DNs) except DN1	(32)
Treg	Foxp3-mRFP	Peripheral lymph nodes: marked CD4^+^CD25^+^ thymocytes and peripheral T cells, lymphocytes, CD4^+^ T cells (7%), B220^+^ B cells or CD8^+^ T cells (<1%) Bone marrow: 12% of CD4^+^ cells	(33)
	Foxp3-GFP	Marked about 90% of CD4^+^CD25^+^ T cells, activated CD44^+^CD62L^-^ cells and CD4^+^ lymphocytes	(34)

## 3 Optical imaging windows

**Figure 2:**
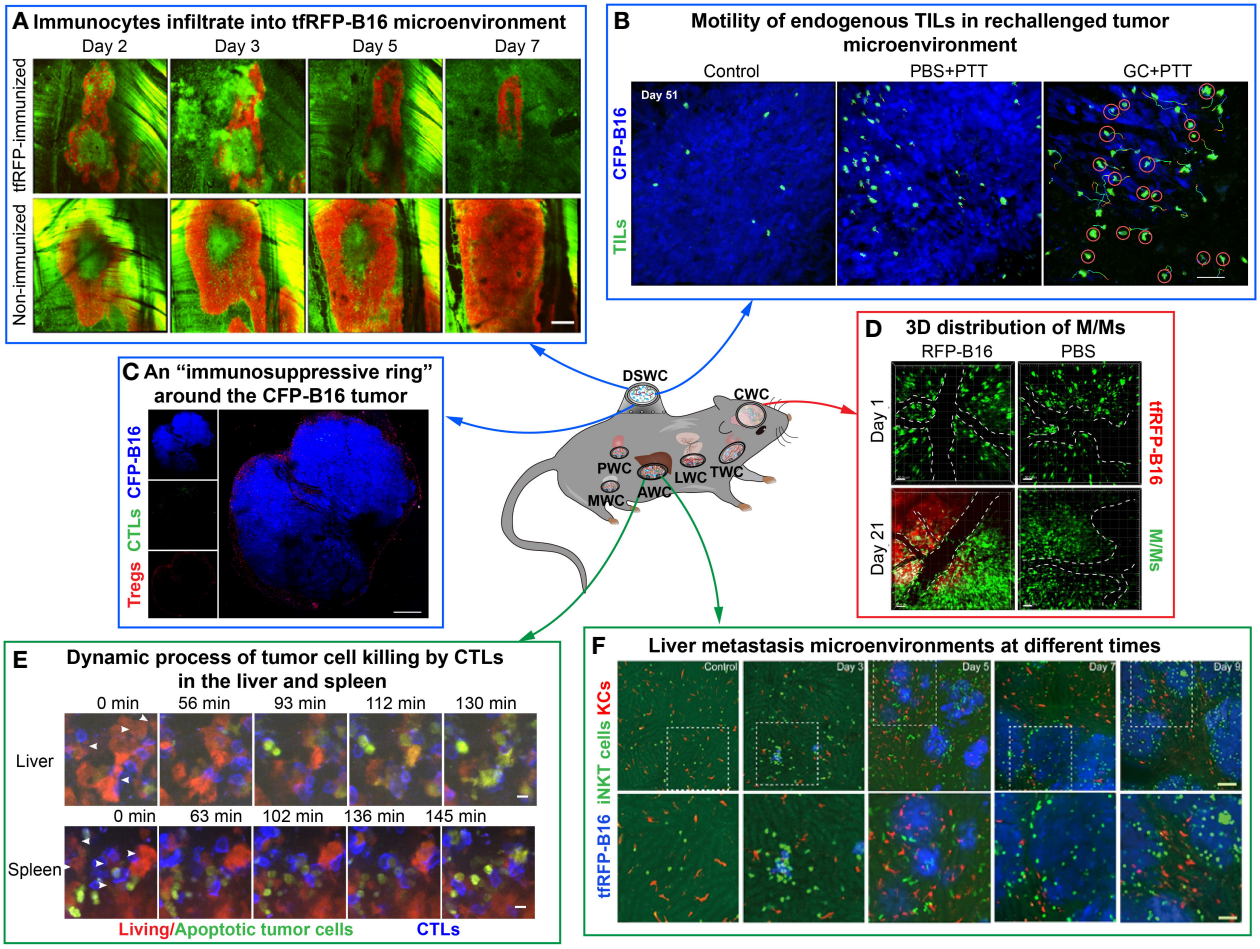
Examples of using optical imaging window mouse models to explore dynamic information in the TME *in vivo*. The figure compiles images from different studies illustrating the dynamic information of immune cells or tumor cells by intravital imaging. **(A–C)** are imaging results of the dorsal skinfold window chamber. **(A)** The tfRFP-B16 microenvironment on certain days. Green: EGFP^+^ host immunocytes; red: tfRFP-B16 cells. Scale bar: 500 μm (35). **(B)** The behavior of endogenous GFP^+^ TILs in the B16 TME *in vivo*. Blue: CFP-B16 tumor; green: Endogenous T cells. Scale bar: 70 μm (15). **(C)** Tregs form a ring around tumor cells. Blue: CFP-B16 tumor; red: Tregs (Foxp3-mRFP cells); green: CFSE-labeled CTLs. Scale bar: 500 μm (5). **(D, E)** are imaging results of the abdominal window chamber. **(D)** The dynamic process of CTLs eliminating tumor cells in different organs. Blue: CTLs; red: living tumor cells; green: apoptotic tumor cells. Scale bar: 10 µm (14). **(E)** Liver metastasis at different time points. Blue: tfRFP-B16 tumor metastases; green: GFP-labeled iNKT cells; red: nanopomegranate-labeled Kupffer cells. The bottom row (scale bar: 50 µm) is an enlargement of the image in the white box in the top row (scale bar: 100 µm) (36). **(F)** is the imaging result of the cranial window chamber. **(F)** Representative data on the 3D distribution of macrophages/monocytes (M/Ms) on day 1 and day 21 post-injection of RFP-B16 cells or PBS. The white dotted line represents the blood vessel. Red: tfRFP-B16 cells. Green: M/Ms. Scale bar: 50 μm (37).

With the development of intravital imaging technology, imaging windows for different murine organs have emerged to improve imaging clarity and stability. The establishment of imaging windows for mouse brain, thyroid, lung, pancreas, liver, mammary and other organs is now relatively mature. Their combination with high-resolution microscopy and fluorescent labeling techniques offers the possibility to observe long-term and dynamic changes in the TME. Imaging windows have offered researchers insights into the processes and mechanisms of tumor development. In this section, we describe the location and function of each imaging window (**Figure 2**). Researchers can select the appropriate imaging window according to the location of the tumor or tumor metastases and the window descriptions.

### 3.1 Dorsal skinfold window chamber

Dorsal skinfold window chamber (38, 39) is the most commonly used imaging window to observe TME, which located in the skin of the mice (**Figures 2A–C**). The surgical procedure for the preparation of a skinfold window is that implant titanium window frames on the back of the mouse and open the skin on one side, and then implant tumor cells into the window. By using the DSWC, intravital imaging of TME for several weeks can be achieved. Scientists utilized this model to observe the anti-tumor immune response within the microenvironment of various murine tumors, such as melanoma (B16-F0 and B16-F10 cell lines) (5, 35), colon cancer [CT26 (22, 40) and MC38 cell lines (41)], breast cancers (4T1 cell lines), etc.

### 3.2 Abdominal window chamber

Abdominal window chamber (30–34) is opened in the abdomen of mice and enables long-term dynamic imaging of TME (**Figures 2D, E**). To solve the problem of poor imaging quality due to animal breathing shakes in the traditional abdominal window, scientists developed a drawer-type abdominal window model called DAWarc, which incorporates an acrylic/resin cover. It can effectively reduce the impact of respiratory jitter on the imaging quality and realize both photoacoustic and fluorescent imaging on the same mouse. This innovative model facilitates intravital imaging, providing valuable insights into immune cell dynamics and the hepatic lobule structure in various liver diseases (42). Additionally, utilizing the DAWarc, researchers visualized the elevated levels of reactive oxygen species (ROS) and caspase-3 activation in specific circulating tumor cells in the liver. These findings suggest that elevated ROS and activated caspase-3 may serve an essential function in the cleavage of gasdermin E proteins (GSDME) and the perforation of plasma membrane (13). Abdominal window chamber can be used to study the primary tumor microenvironment or tumor metastases in abdominal organs such as the liver (42, 43), spleen (14), and colon (44) *in vivo*.

### 3.3 Cranial window chamber

Cranial window chamber (45) is located at the mouse cranium and serves as a powerful window for long-term monitoring the brain microenvironment (**Figure 2F**). Professor Zhang’s team visualized the dynamic information of microglia in the brain metastasis of melanoma over a long time through the bilateral cranial window model of transgenic mice CX_3_CR_1_-GFP. Long-term morphodynamical changes in microglia during the development of melanoma brain metastases were monitored by intravital imaging (37). Cranial window chamber allows for observing glioma microenvironment in real time, as well as the microenvironment of brain metastases of various malignancies (eg, melanoma (37), breast cancer (46), lymphoma (47), etc.).

### 3.4 Mammary window chamber

Mammary window chamber (48) was developed and positioned at the top of the mammary gland of mice. Mammary window chamber enables long-term and high-resolution visualization of the breast TME. Researchers observed that macrophages can enhance vascular permeability through VEGFA signaling in the mammary TME. Then, the tumor cells migrated toward TME metastasis at sites of transient permeability of vessels (49). Besides, a subtype of dendritic cells (DCs) was demonstrated that they could promote T-cell interactions in breast cancer through the mammary window chamber (50).

### 3.5 Pancreas window chamber

Pancreas window chamber (51, 52) is a window specifically designed for pancreatic tumor imaging and can be used for long-term imaging of the pancreas. Researchers used abdominal window for imaging of pancreatic tumors previously (53). The lateral and medial stability is poor due to bowel movements and respiration. The pancreas window increases lateral and medial stability and significantly improves the imaging quality. Several patterns of tumor cell motility were visualized through pancreas window chamber, including tumor cell collective migration and single cell migration (52). Researchers also observed that vascular contents extravasated through transient vascular opening and were then rapidly cleared (52).

### 3.6 Lung window chamber

Lung window chamber (54) is a minimally invasive technique that involves creating a window between the 6th and 7th ribs of a mouse’s chest. Lung window chamber allows imaging of the same lung tissue within a few weeks and the repositioning of the same cells (54). Recent studies have observed that disseminated breast tumor cells extravasated rapidly from the vascular and had high survival rates in the lung metastatic TME (55). Lung window chamber enables high-resolution and long-term imaging of the mouse lung TME *in vivo*.

### 3.7 Thyroid window chamber

Thyroid window chamber (56) is mounted on the neck of mice to visualize the dynamic information in the thyroid TME. Due to the specific anatomical location of the thyroid gland, researchers applied the cross-stitch technology in the thyroid window, which was suitable for the small thyroid tumor. Thus, researchers realized high-resolution, multi-day intravital imaging of the mouse thyroid TME. Recent studies intravitally observed that the thyroid TME was abundant in macrophages and neovascularization, and only a few macrophages showed strong motility (56).

## 4 Imaging data analysis

After acquisition of intravital images, it is necessary to do the further processing and analyze the imaging data. Through quantitative parameters, the dynamic behavior and infiltration of immune cells in the TME can be quantitively assessed, further revealing their biological characteristics and immune functions. Researchers could infer whether the tumor microenvironment is in an immune-suppressive or immune-activated status according to the imaging data results. The relevant processes and methods of imaging data analysis are summarized based on the experience of previous works belonged to Professor Zhang’s team. Besides, we further related the obtained parameters to the anti/pro-tumor function of immune cells.

### 4.1 The process of imaging data analysis

(1) Data preprocessing: The collected spatio-temporal imaging data were subjected to image preprocessing by using ImageJ/Fiji (NIH, USA) or Imaris software (Oxford Instruments, UK). A series of preprocessing methods including denoising, correction and enhancing the contrast were applied to enhance the quality of the images. In cases of channel crosstalk, spectral decomposition was employed to eliminate the interfering signals between different channels. Subsequently, MATLAB software (MathWorks, USA) was utilized for image segmentation to extract regions of interest (ROI).
(2) Key parameters extraction: The imaging data were imported into Imaris software for direct observation of cell morphology, cell density and spatial distribution. Furthermore, we can calculate the contact time between immune cells and tumor cells in Imaris software. The “spots” function of Imaris software was used to facilitate statistical analysis of cell density and distribution, and tracking of cell movement trajectories. It should be noted that, during the cell tracking, careful threshold adjustments needed to be made to filter the generated tracks. Manual tracking was performed for the cells when the automatic tracking failed or went wrong. Contact time was determined by defining no contact between immune cells and tumor cells as 0 min, and then counting the time that each immune cell was in junction with a tumor cell. All key parameters were exported into the Excel table format, followed by separation of parameters.
(3) Data presentation: The table containing trajectory data table was imported into MATLAB software to generate the trajectory with normalized origin and average displacement curves. The key parameters such as mean velocity, arrest coefficient and arrest coefficient were also imported into GraphPad Prism software (GraphPad Software, USA) for significance analysis. The dot plots were drawn to present the analysis results and each dot represents a cell. Imaris software was employed to generate 3D spatial images and make dynamic videos, enabling real-time visualizing of cellular dynamics.

**Figure 3:**
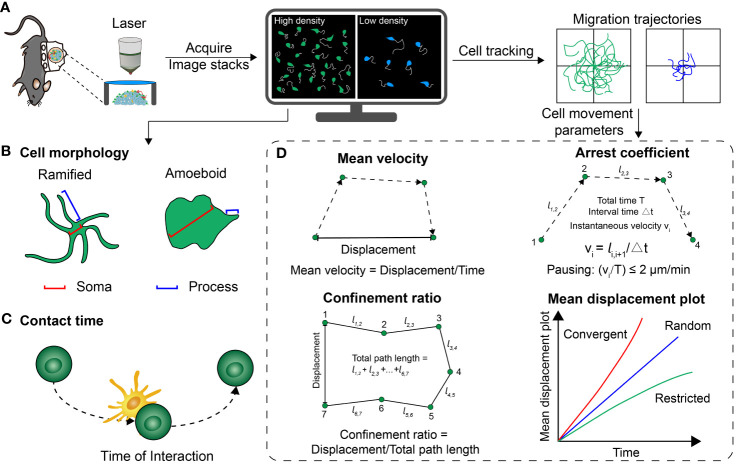
Several common parameters that quantitatively analyze the cell distribution, morphology and motility behavior. **(A)** Basic procedure for acquiring optical imaging data *in vivo*. **(B)** Schematic representation of the different morphologies of cells. **(C)** Schematic of the contact time between two cells. **(D)** A series of parameters extracted from the trajectory of cell movement, including mean velocity, confinement ratio, arrest coefficient and mean displacement plot.

The above is the process of processing and analyzing imaging data after it has been acquired (**Figure 3**) (57). Next, we will introduce the definition and immune function of key parameters.

### 4.2 Cell density

Cell density refers to the quantification of immune cells recruited to the TME, measured as the number of cells per unit area. Dr. Shuhong Qi et al. performed intravital imaging on the B16 melanoma microenvironment during the cyclophosphamide (CTX) combined adoptive cell therapy (ACT). In the TME treated with combined CTX-ACT, it was found that the density of endogenous cytotoxic T cells (CTLs) (119.2 cells/mm^2^) was significantly lower compared to mice treated with ACT alone (411.9 cells/mm^2^). Intravital imaging showed that the combined treatment of CTX-ACT effectively eliminated a significant portion of immunosuppressive T cells within the tumor region. Additionally, the activated CTLs were observed to localize and efficiently engage in the eradication of tumor cells (5). By quantifying the density of immune cells infiltrating in the TME, we can determine whether the tumor is “cold” or “hot” or it can suggest a certain stage of immunotherapy, which helps to understand the immune status of the tumor, and guide the tumor immunotherapy.

### 4.3 Cell morphology

The morphology of immune cells varies between their resting and activated states. The cell morphology serves as indicator of their activation status, enabling the determination of their functional state based on these observable changes in cell structure. In a previous study examining the development of melanoma brain metastases, the activation state of microglia/macrophages was determined by defining a branching parameter. This parameter was defined as the ratio of the mean soma diameter to the mean process length. When the branching parameter increased beyond 0.5, the cells were classified as ramified indicating an enhancement of activation; when the parameter decreased approach 0.5, the cells were defined as amoeboid, indicating a resting state. Quantitative analysis revealed that the branching parameters of microglia/macrophages in the tfRFP-B16 group (melanoma brain metastases) were significantly higher compared to the phosphate-buffered saline (PBS) control group. It indicated that microglia/macrophages in the tumor area were activated and performed a pro-tumor function (37). By quantitatively characterizing the morphological changes in immune cells, their activation status in the TME can be described accurately and correlate its activation status with anti/pro-tumor function.

### 4.4 Contact time

Contact time indicates the duration of the interaction between two cells. Professor Zhang’s team visualized the dynamic process of tumor cell killing by CTLs and found that several CTLs closely surrounded the tumor cells in the liver metastasis. These CTLs induced apoptosis (programmed cell death) of the tumor cells in more than 1 hour. In the B16 expressing ovalbumin protein and caspase-3 fluorescence resonance energy transfer (FRET) probe, the time of each CTL contacting with tumor cells was 49.4 minutes. This was much longer compared to the contact time of 3.9 minutes observed in the B16 cells expressing only the caspase-3 FRET probe. These findings indicated that the cumulative contact time can promote the apoptosis of tumor cells (14). In the TME, contact time can be employed to assess the killing efficacy of immune cells such as CTLs (29), NKTs (58), macrophages (59), and neutrophils (34) on the tumor cells, reflecting the anti-tumor functions of immune cells.

### 4.5 Mean velocity

Mean velocity is one of the quantitative parameters to reflect cell migration, which measures the migratory speed. In the study of cellular dynamics of adoptive T cells using intravital imaging, trajectory tracking was conducted to monitor the movement of T cells in the TME. This analysis revealed that T cells located in collagen-dense areas within the tumor periphery exhibited higher mean velocities (4.3 μm/min) compared to those in the tumor core (1.4 μm/min). After anti-PD-L1 monotherapy, T cells in these two locations exhibited slower migration speed (1.8 μm/min) compared to the control group (4.6 μm/min). This suggested that T cells were more effective in targeting and eliminating tumor cells (60). The heterogeneity in the mean velocity of immune cell migration in different regions and at different time points could represent the activation status and anti-tumor function of immune cells.

### 4.6 Arrest coefficient

Arrest coefficient is determined by the time when the instantaneous velocity of the cell is below a certain value compared to the total time, which provides insight into the state of cellular stagnation. In the context of cell migration, when a cell’s speed is below 2 μm per minute, it is often defined as “pausing” (61). In a study conducted by Professor Zhang’s team, intravital imaging was employed to observe tumor infiltrating lymphocytes (TILs) in the rechallenged B16 melanoma *in vivo* in the N-dihydrogalactochitosan (GC) + photothermal therapy (PTT), PBS + PTT and control groups. The TILs in the GC + PTT group had the lowest arrest coefficient (GC + PTT: 44 ± 34%, PBS + PTT: 91 ± 19%, Control: 90 ± 20%), which indicated that TILs were more active in the GC + PTT group, resulting in increased infiltration of endogenous TILs for tumor cell elimination (15). By quantifying the rate of cell movement, arrest coefficient could suggest the state of cellular stagnation, which indicates the tumor cell killing ability.

### 4.7 Confinement ratio

The confinement ratio is a parameter that measures the degree of cell movement restriction within certain time. It is calculated by dividing the displacement of cells by the total distance covered by the cells during that specific time (61). Professor Zhang’s team previously visualized the motility of invariant natural killer T (iNKT) cells in the liver metastases and found that iNKT cells infiltrating into the tumor parenchyma (Confinement ratio: 0.19) were significantly less motile and had more restricted trajectories compared to the peripheral tumor (0.24), out of tumor (0.39) and control (0.40). This spatial heterogeneity in the confinement ratios of iNKT cells indicated their activation and interaction with tumor cells in the TME (36). The combination of mean velocity, arrest coefficient and confinement ratio are usually used to describe the motility of immune cells more accurately and quantitatively, to assess the activation status and tumor cell killing ability of immune cells in the TME.

### 4.8 Mean displacement plot

The mean displacement plot is the square root of the displacement divided by time. When the curve shows a linear correlation, it indicates that the cells are mainly engaged in random free movement; when the curve bends upward, it indicates that the cells are in the convergent movement; when the curve bends downward, it indicates that the cells are mainly in the restricted movement (61). The mean displacement plot can be used to evaluate the movement of immune cells in the tumor region (22). The previous study investigated the migration behavior of adoptive CTLs in the TME *in vivo*, and its mean displacement plot indicated that the adoptive CTLs exhibited a random walk feature after the CTX-ACT treatment (5). The mean displacement plot reflects the motor behavior of immune cells, which is closely related to their anti-tumor function.

## 5 Intravital imaging for the functions of immune cells during the immunotherapy

**Figure 4:**
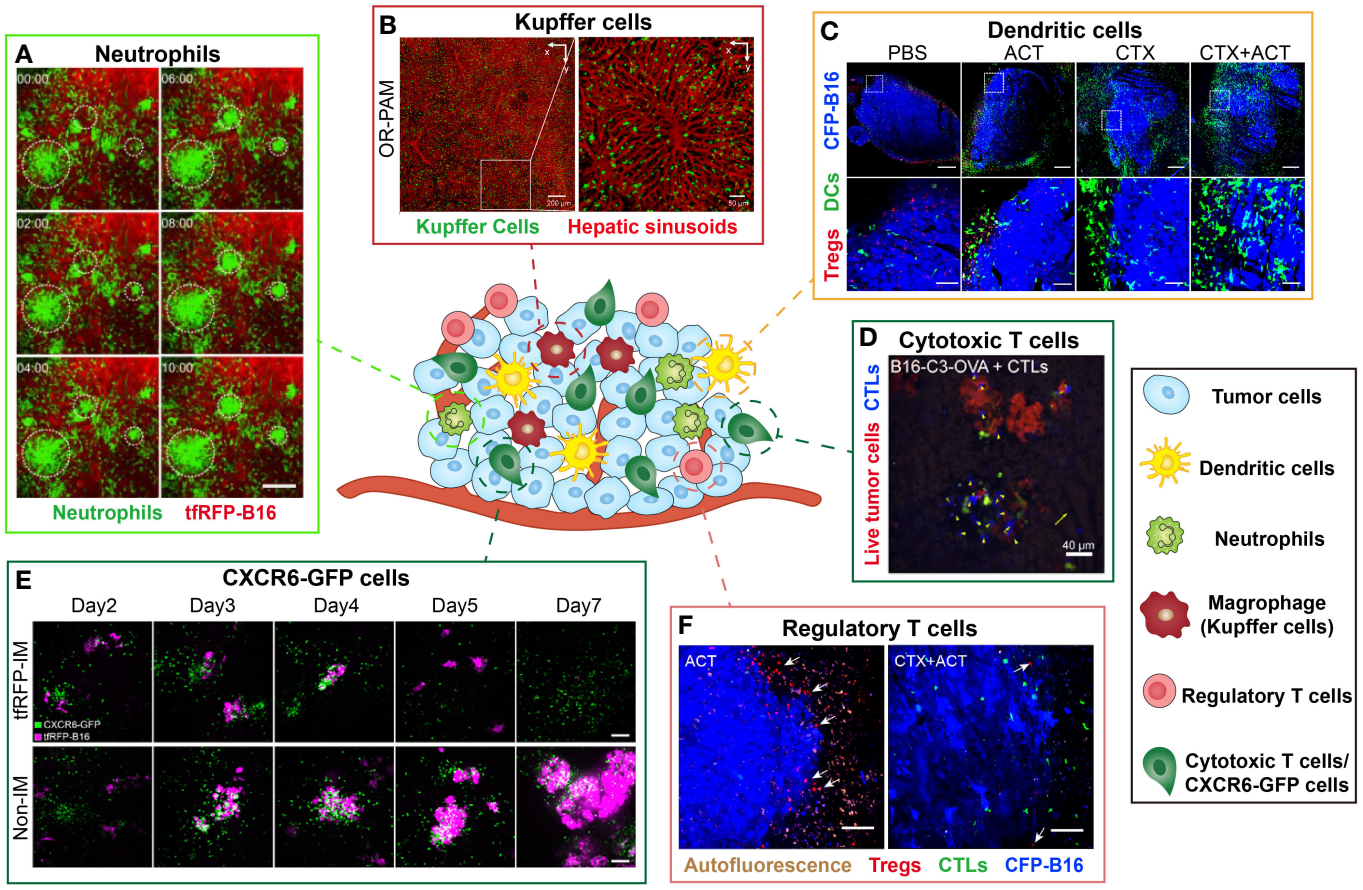
Examples of intravital imaging applications to illustrate functions of immune cells during tumor immunotherapy. The figure compiles images from different studies exploring the distribution and function of immune cells *in vivo*. Images are representative of **(A)** Imaging results of cell aggregates formed in tfRFP-immunized mice at different time. Green indicates Ly6G^+^ neutrophil and red represents tfRFP-B16 tumor. Scale bar: 100 μm (35). **(B)** By the optical-resolution photoacoustic microscopy system, intravital imaging of hepatic sinusoids and KCs through dual-wavelength photoacoustic mapping after intravenous injection. Red represents hepatic sinusoids and green represents KCs. Scale bar: 200 μm (left), 50μm (right) (66). **(C)** Imaging results of DCs and Tregs in the CFP-B16 TME in different groups. Blue represents CFP-B16 tumor, red represents Tregs and green indicates DCs. The bottom row (scale bar: 100 µm) is an enlargement of the image in the white box in the top row (scale bar: 500 µm) (5). **(D)** Images of a representation of CTL displacement and the distribution in liver metastasis. Blue represents CTLs and Red represents living tumor cells. Scale bar: 40 µm. Arrows indicate CTLs displacement (14). **(E)** Imaging results of the tfRFP-B16 liver metastases in the CXCR6GFP/+ mice from Day 2 to Day 7. The top row corresponds to a tfRFP-immunized mouse, while the bottom row corresponds to a non-immunized mouse. Magenta indicates tfRFP-B16 tumor and green represents CXCR6-GFP cells. Scale bar: 100 µm (13). **(F)** Imaging results of Tregs at the CFP-B16 tumor margins in CTX and CTX+ACT group. Red represents Tregs, green represents CTLs and blue indicates CFP-B16 tumor. Scale bar: 100 µm. The arrows indicate Tregs (5).

The TME is composed of tumor cells, immune cells, blood vessels, fibroblasts, and extracellular matrix. Immune cells have a vital impact on the tumor immunosurveillance or immunosuppression during immunotherapy. Intravital imaging offers a valuable approach to studying the dynamic behavior of immune cells within the TME at the single-cell level, allowing long-term observation of the spatiotemporal dynamics of multiple immune cells and their interactions with tumor cells. This facilitates our understanding of immunotherapeutic mechanisms and helps improve the immunotherapy. We review the dynamic processes of various immune cells in the TME and their role in immunotherapy *in vivo* (**Figure 4**).

### 5.1 Tumor-associated neutrophils

Neutrophils are a type of white blood cells that help the body defend against infections. Intravital imaging allows direct observation of the neutrophil anti/pro-tumor functions. Professor Zhang’s team used intravital imaging and skinfold windows to observe that the neutrophils mediated anti-tumor immune response in the tfRFP-B16 melanoma microenvironment (**Figure 4A**). Imaging results illustrated that neutrophils were recruited to the tumor region, aggregated into clusters in the TME, and contributed to the effective killing of tumor cells (35). Intravital imaging also revealed that tumor-associated neutrophils (TANs) tend to be associated with poor prognosis. The investigators observed that tumor cells induced the formation of neutrophil extracellular traps through intravital imaging, and the formation of neutrophil extracellular traps promoted the migration and invasion of tumor cells (62).

### 5.2 Tumor-associated macrophages

Tumor-associated macrophages (TAMs) are the most abundant group of immune cells and demonstrate anti/pro-tumor function in the TME. An intravital imaging research illustrated that the interaction between macrophages and tumor cells enhanced vascular permeability and promoted tumor cell intravasation in mammary tumors (63). Another intravital imaging study also demonstrated that TAMs in MC38 colon TME would capture anti-PD-1 mAb, making it fail to effectively bind PD-1^+^ tumor-infiltrating CD8^+^ T cells (41). Scientists also visualized that in the liver B16-F10 metastases, liver macrophages eliminated circulating tumor cells through antibody-dependent phagocytosis way (64). Intravital imaging helps reveal the bidirectional regulatory role of TAMs in the tumor immune response.

### 5.3 Microglia

Microglia, as the most important immune cells in the central nervous system, maintain their homeostasis and participate in the occurrence and development of brain tumors. One of our studies revealed that microglia played a role in promoting the process of tumor brain metastasis. Intravital imaging results showed that large numbers of microglia were recruited in the melanoma metastases and that microglia changed morphologically from ramified to an amoeboid shape. The result suggested that microglia were activated to promote brain metastasis of melanoma (37). In addition, investigators observed microglia interactions with tumor vasculature *in vivo*. The anti-vascular treatment revealed that the reduction in tumor vasculature was accompanied by a significant reduction in microglia, and microglia in the perivascular region showed higher activity (65). Intravital imaging assesses the activation status of microglia by visualizing their morphological changes, which in turn correlates the microglia function with tumor development.

### 5.4 Kupffer cells

Kupffer cells (KCs) are specialized tissue macrophages, constituting a significant proportion of the overall population of fixed macrophages in the body. These cells, which are primarily found in the hepatic sinuses, represent the largest subtype of macrophages and play a vital role in immune surveillance. A previous study developed a nanoparticle, which was used to selectively label KCs. It was observed that KCs strategically distribute along the central vein-portal triad axis in each liver lobule by fluorescence/photoacoustic imaging (**Figure 4B**) (66). Besides, it was also found that the spatial distribution of KCs was closely related to their phagocytic ability. This study showed that the spatial distribution of KCs in the liver was closely related to their phagocytic functions.

### 5.5 Dendritic cells

DCs exert critical effect on the tumor immune response, mainly responsible for presenting antigens and priming T cells. On the other hand, they may also play a role in promoting tumor development. Previous intravital imaging studies have shown that tumor DCs in the murine MCA101 tumor parenchyma formed a network, that trapped T cells and limited their antitumor effectiveness (67). Another intravital imaging study indicated that the DCs at the edge of the murine spontaneous breast tumor interacted with tumor cells and T cells for a long time, but failed to stimulate anti-tumor T cells (68). In the study of combined immunotherapy for B16 melanoma, researchers observed that CTX treatment effectively promoted the infiltration of mature DCs to improve the anti-tumor effect of combined immunotherapy *in vivo* (**Figure 4C**) (5). The distribution of DCs and the interaction between DCs and other immune cells or tumor cells can be visualized by intravital imaging, thus characterizing the anti/pro-tumor function of DCs in the tumor region.

### 5.6 Natural killer cells

Natural killer cells (NKs) can quickly kill tumors without specific antigen stimulation, thus playing a critical role in the tumor immune surveillance. When studying the rapid killing effect of tumor cells by NKs, intravital imaging can visualize the entire killing process, thus evaluating the killing time and efficiency. Philippe Bousso’s team monitored the NKs motility behavior within the tumor by intravital imaging and quantified the difference between NKs and T cells movement behavior during the tumor elimination. The results indicated that T cells established stable and a long time contact with tumor cells for effective killing, while NKs formed a transient and dynamic contact with tumor cells before effective killing (69). These studies revealed that intravital imaging has great advantages in studying the dynamic process of interaction between NK cells and tumor cells, revealing the high-efficient tumor killing effect of NKs.

### 5.7 Tumor infiltrating lymphocytes

TILs are one of the important components of TME, and have distinct cell subsets. The intravital imaging study of TILs has shown that during the process of tumor regression, TILs migrated randomly in the tumor periphery. After entering the tumor, TILs had long-term contacts with tumor cells and macrophages to elicit effective anti-tumor response (70). Besides, our previous research used laser immunotherapy to generate immune memory against tumor cells. By employing intravital imaging to observe the TME at rechallenging sites of laser immunotherapy-treated mice, we observed an increase in the infiltration of TILs with active motility. Approximately half of the TILs arrested and had long-lasting interactions with tumor cells and then eliminated tumor cells (15). Through intravital imaging, it is possible to study the movement behavior of TILs in the TME, as well as their interactions and contacts with tumor cells and immune cells, to investigate their anti-tumor effects.

### 5.8 Cytotoxic T lymphocytes

CTLs are instrumental in the anti-tumor immune response, thus a lot of intravital imaging studies have focused on the dynamic information and function of CTLs in the TME. The spatial distribution, movement behavior of CTLs and interaction with CTLs in the tumor areas have been visually revealed. It is reported that during the process of tumor clearance, the movement behavior of CTLs was divided into three stages: migrating at high speeds, arresting to contact with tumor cells, and then resuming movement (71). Intravital imaging provided an effective tool for visualizing the movement characteristics during these three stages. During the investigation of CTX combined with ACT treatment for B16 melanoma *in vivo*, Professor Zhang’s team observed dynamic changes in the TME. The migratory behavior of the adoptive CTLs differed between the tumor periphery and parenchyma. Unlike the complex migratory behaviors of adoptive CTLs, the endogenous CTLs displayed an arresting behavior within the tumor area, engaging in interacting with tumor cells and eliminating them (5). By intravital imaging, the researchers quantitatively compared the killing ability of adoptive CTLs against metastatic tumor cells in liver and spleen, and found that the killing ability of CTLs against tumor cells was significantly reduced due to the immunosuppressive liver microenvironment (**Figure 4D**) (14). Intravital imaging can visualize the movement behavior of CTLs and monitor the interaction between CTLs and tumor cells in the TME which is closely linked to their tumor cell killing ability (**Figure 4E**) (13).

### 5.9 Regulatory T cells

Regulatory T cells (Tregs) are crucial for immunosuppression in the TME. Recently, several studies employed intravital imaging techniques to investigate the spatial location and immune function of Tregs within the TME during immunotherapy. The researchers found that the majority of Tregs migrated at high speeds both in CT26 tumor parenchyma and the surrounding tumor stroma, and only transiently contacted with antigen presenting cells by intravital imaging (40). Our previous research observed that Tregs formed an “immunosuppressive ring” around a murine B16 melanoma tumor to prevent adoptive CTLs from entering the tumor region. When the CTX treatment depleted Tregs and blocked the “immunosuppressive ring”, adoptive CTLs can effectively infiltrate into the solid tumor and then effectively killed the tumor cells (**Figure 4F**) (5). Intravital imaging can visualize the spatial distribution of Tregs in the TME which is closely linked to their immune-inhibiting function (**Figure 4F**) (5). Furthermore, the intravital imaging also revealed that Tregs suppressed anti-tumor immune responses by contact with other immune cells (40).

### 5.10 Chimeric antigen receptor T-cells

The utilization of T cells that express chimeric antigen receptors has demonstrated remarkable effectiveness in the treatment of systemic B cell malignancies. Philippe Bousso’s team used intravital single-cell imaging to reveal the activity and killing function of chimeric antigen receptor T (CAR-T) cells in the TME (72) and a cross-talk between CAR-T cell subsets (73). The intravital imaging results showed that, in the bone marrow (the TME of B cell malignancies), CAR-T cells destroyed their targets within 25 min (72). Besides, scientists have visualized primary central nervous system lymphoma growth, dynamic behavior and therapeutic effects of CAR-T cells in the same mouse over several weeks by intravital imaging (47). By visualizing the dynamic process of interaction between CAR-T cells and target cells in real time, intravital imaging helps to assess the killing effect of CAR-T cells on tumor cells, thus revealing the role of CAR-T cells in the anti-tumor response in the TME.

## 6 Discussion and conclusion

Intravital imaging has found widespread application in cancer research, which also can be applied to other disease researches, such as autoimmune diseases [multiple sclerosis (16), rheumatoid arthritis (74)], neurologic diseases (75, 76), cardiovascular diseases (77, 78), etc.). Especially in the autoimmune diseases study, intravital imaging allows for the visualization of immune cell migration and functions, and other critical processes such as drug delivery and distribution *in vivo*. Professor Zhang’s group visualized that one kind of nanomedicine (named Cur-HPPS) can specifically inhibit the infiltration of inflammatory monocytes into the mouse central nervous system in the experimental autoimmune encephalomyelitis (EAE) mice through cranial window (16). Another group applied the fluorescent dye labelling drug (AF647-CTLA-4 Ig) into the treatment of mouse rheumatoid arthritis (74). Intravital imaging revealed that AF647-CTLA-4 Ig began to drain through the lymphatic vessels after injection and then arrived at inflammation site. Furthermore, intravital imaging also enables the observation and analysis of autoimmune disease processes, such as inflammation reactions, spatial distribution and migration of immune cells, deepening our understanding of autoimmune disease mechanisms.

Intravital imaging serves as a powerful tool for investigating the movement behavior and function of immune cells, as well as their anti-tumor immune response within the TME. With immunotherapy emerging as a highly promising approach in cancer treatment, intravital imaging holds great potential in providing spatio-temporal dynamic information regarding immunotherapies *in vivo*. This review explores various imaging window models, immune cell labeling techniques, and intravital imaging methods employed to elucidate the roles played by innate and adaptive immune cells in the TME during immunotherapy. While intravital imaging has provided a long-term and dynamic view of the interplay between the immune system and solid tumors, the demands for intravital imaging technology are continuously evolving and becoming more diverse. The researchers have developed 4D intravital imaging system to obtain immune landscapes *in vivo* (79). Moreover, when intravital imaging is combined with some new techniques, such as single-cell transcriptome and proteome, spatial transcriptome and multi-staining immunofluorescence, they are expected to provide more information, linking single immune cell behaviors with phenotypic, transcriptomic, genomic and proteomic features. These novel methodologies hold the potential to serve as unique and indispensable tools for studying the complex and dynamic interactions between immune cells and tumor cells. These technologies also facilitate the evaluation of immunotherapy *in vivo* and contribute to the development of new immunotherapeutic approaches.

## Author contributions

XP: Conceptualization, Formal analysis, Investigation, Methodology, Visualization, Writing – original draft, Writing – review & editing. YW: Formal analysis, Visualization, Writing – review & editing. JZ: Formal analysis, Writing – review & editing. ZZ: Funding acquisition, Project administration, Supervision, Resources, Writing – review & editing. SQ: Conceptualization, Funding acquisition, Formal analysis, Data curation, Supervision, Resources, Investigation, Writing – original draft, Writing – review & editing.
